# Potential drug-drug and drug-disease interactions in prescriptions for ambulatory patients over 50 years of age in family medicine clinics in Mexico City

**DOI:** 10.1186/1472-6963-7-147

**Published:** 2007-09-19

**Authors:** Svetlana Vladislavovna Doubova (Dubova), Hortensia Reyes-Morales, Laura del Pilar Torres-Arreola, Magdalena Suárez-Ortega

**Affiliations:** 1 Epidemiology and Health Services Research Unit. National Medical Center Century XXI, Mexican Institute of Social Security, Mexico City, Mexico

## Abstract

**Background:**

In Mexico, inappropriate prescription of drugs with potential interactions causing serious risks to patient health has been little studied. Work in this area has focused mainly on hospitalized patients, with only specific drug combinations analyzed; moreover, the studies have not produced conclusive results. In the present study, we determined the frequency of potential drug-drug and drug-disease interactions in prescriptions for ambulatory patients over 50 years of age, who used Mexican Institute of Social Security (IMSS) family medicine clinics. In addition, we aimed to identify the associated factors for these interactions.

**Methods:**

We collected information on general patient characteristics, medical histories, and medication (complete data). The study included 624 ambulatory patients over 50 years of age, with non-malignant pain syndrome, who made ambulatory visits to two IMSS family medicine clinics in Mexico City. The patients received 7-day prescriptions for non-opioid analgesics. The potential interactions were identified by using the Thompson Micromedex program. Data were analyzed using descriptive, bivariate and multiple logistic regression analyses.

**Results:**

The average number of prescribed drugs was 5.9 ± 2.5. About 80.0% of patients had prescriptions implying one or more potential drug-drug interactions and 3.8% of patients were prescribed drug combinations with interactions that should be avoided. Also, 64.0% of patients had prescriptions implying one or more potential drug disease interactions. The factors significantly associated with having one or more potential interactions included: taking 5 or more medicines (adjusted Odds Ratio (OR): 4.34, 95%CI: 2.76–6.83), patient age 60 years or older (adjusted OR: 1.66, 95% CI: 1.01–2.74) and suffering from cardiovascular diseases (adjusted OR: 7.26, 95% CI: 4.61–11.44).

**Conclusion:**

The high frequency of prescription of drugs with potential drug interactions showed in this study suggests that it is common practice in primary care level. To lower the frequency of potential interactions it could be necessary to make a careful selection of therapeutic alternatives, and in cases without other options, patients should be continuously monitored to identify adverse events.

## Background

During the last decades in Mexico, as elsewhere, the population has aged, causing an increase in the level of chronic degenerative diseases and a consequent increment in medication. Polypharmacy is now common, and carries a high risk of drug-drug interactions and drug-disease interactions. These may cause adverse effects, or the therapeutic effects of the combined medicines may change, with serious consequences for health. In the United States 25% of ambulatory patients taking drug combinations were at risk for clinically important interactions [1]. Furthermore, it has been reported that about 40% of hospitalized patients had at least one potential drug-disease interaction [2]; a large study including 70,203 outpatient visits by patients aged 65 and older found that 2.6% ofvisits with at least one prescription had one or more of the 50 inappropriate drug-disease combinations examined [3]. A European study of 1601 ambulatory elderly patients, taking an average of seven different drugs, found that 46.0% were at risk for at least one clinically important potential drug-drug interaction [4]. It has been shown that inappropriate prescription combinations increase with patient age, are more frequent in men, rise in line with the Charlson co-morbidity index [5], and increase as the number of prescribed drugs increases [2,3,6-8]. It is possible that other risk factors for potential interactions exist, and these should be identified to establish successful methods for improving prescription practices.

Adverse consequences of drug interactions have been shown in various studies. Drug-drug interactions cause 4.8% of hospitalizations attributed to drugs in the elderly [9-11]. In most cases they are erroneously interpreted as patient deterioration because of illness, poor adherence to prescribed treatment, or infection [12].

In Mexico, inappropriate prescription of drugs with potential interactions causing serious risks to patient health has been little studied. Work in this area has focused mainly on hospitalized patients, with only specific drug combinations analyzed [13]; moreover, the studies have not produced conclusive results [14,15].

In the present study, we sought to determine the frequency of potential drug-drug and drug-disease interactions in prescriptions of ambulatory patients over 50 years of age, who attended to Mexican Institute of Social Security (IMSS) family medicine clinics. In addition, we aimed at identifying factors associated to them.

## Methods

The present study is a secondary data analysis from the Educational Strategy Study (ESS) involving both doctors and patients over 50 years of age, focused on improving utilization of non-opioid analgesics in primary care study and carried out during 2006 in two large IMSS family medicine clinics (FMC) located in Mexico City.

IMSS is the largest medical institution in Mexico that provides health care for more than 53 million of Mexicans. The provision of services is divided into three levels, where the Family Medicine Clinic is the primary care level. IMSS FMC range from 2 to 40 examining rooms; the clinics included in the study had 32 examining rooms, which means 64 family doctors working in the morning and evening shifts (32 in each);. These clinics were selected by convenience and are similar in organization to the rest of those that constitute IMSS primary care system. Cross-sectional data from 624 ambulatory patients over 50 years of age who made visits to 127 family doctors (96% of all family doctors) were collected. On average, five patients seen by each family doctor were included consecutively. The patients studied suffered from non-malignant pain syndrome and received prescriptions of non-opioid analgesics for 7 days or more. The variables analyzed were general characteristics of the patient (age, marital status, literacy), medical history (number of chronic diseases, number of medicines prescribed), and complete information about all oral and injected drugs prescribed by the doctors during the consultation (including occasional drugs and those that were prescribed for regular use, besides the prescription of non-opioid analgesics described as inclusion criteria). All patients were personally interviewed immediately after the visit and the information from the electronic medical record and from the electronic prescription was registered. Most of information (diagnosis and prescriptions) was obtained through personal interview and additional data were obtained from the electronic medical charts and the electronic prescriptions.

The International Classification of Disease, version 10 (ICD-10) [16], and the Anatomical Therapeutic Chemical classification system [17] served to codify the data. To look for potential interactions every combination of prescribed drugs was analyzed by using the Thompson Micromedex program [18]. Drug-drug interactions were sorted by clinical relevance using the Classification System of the Department of Pharmacology, Hospital Huddinge, Stockholm, Sweden [19]. In this classification, drug interactions are rated A and B when they are not of clinical importance (type A), or the effect of the interaction has not yet been established (type B). One interaction of type C can cause possible changes in the therapeutic effects, or may cause adverse effects, but can be avoided adjusting the individual drug doses. A potential drug-drug interaction of type D indicates a potential for severe adverse effects; individual dose adjustment is difficult in these cases. For this paper, only drug-drug interactions of type C and D were detected and analyzed. Using the Zhan Classification all drug-disease interactions were classified as being of low, moderate or high clinical significance [3].

The IMSS Ethics Committee approved the ESS (registration number: 2005-785-185).

### Statistical analysis

All collected data regarding medications prescribed were included in the analysis. The descriptive analysis included absolute and relative frequencies of categorical variables. A bivariate analysis to ascertain potential factors associated with drug-drug and drug-disease interaction was performed using the chi-square test for categorical variables. To evaluate the risk factors related to the presence of drug-drug interactions in prescription mixes, a multiple logistic regression analysis by using the backward stepwise method was performed; the correlation terms and interactions among selected variables were also explored, and goodness of fit test was assessed for the best model. Variables that were explored in the bivariate analysis were: gender (female), marital status (single), age (≥ 60 years of age), literacy (only elementary school o less), number of chronic conditions, diagnosis (cardiovascular, gastrointestinal, endocrine, genital and urinary, neurological, mental, infection disease, as well as musculoskeletal and joint disorders), and number of drugs prescribed (≥ 5). Only statistically significant associations and plausible variables were considered for the logistic regression model.

All *P*-values were obtained from two-tailed tests and the significance level selected was *P *= 0.05. The programs SPSS 10.0 for Windows (SPSS Inc, Chicago, IL), and the statistics package of STATA Corporation (College Station, TX) were used to analyze data.

## Results

The median age of patients was 69 years (range 50–94 years). Most (78.7%) were women, 63.9% were housewives and more than half were single, divorced or widowed. The average number of chronic diseases per patient was 3.4 ± 1.5 and the most frequent illnesses were degenerative joint disease, hypertension, chronic gastritis, diabetes mellitus and dyslipidemia (Table 1). The total number of medicines prescribed to the 624 patients was 3,739 with an average of 5.9 ± 2.5 drugs per patient. The most frequent drugs prescribed were active on the alimentary tract, or affected general metabolism (drugs combating gastrointestinal diseases were the most commonly prescribed drugs). The next most common class of drugs was active on the cardiovascular system; drugs addressing muscle-skeletal system problems were next in prescription frequency, and, finally, drugs active on the nervous system. In this final group paracetamol was the main drug consumed (Table 2).

**Table 1:** General patient characteristics and medical history

**Characteristics**	n = 624 **n (%)**
**General patient characteristics**	
Female	491 (78.7)
Lives without couple (Single, Divorced, Widow)	320 (51.3)
**Occupation**	
Housewife	399 (63.9)
Retired-pensioner	119 (19.0)
Services	16 (2.6)
Unskilled worker	38 (6.0)
Clerk	48 (7.7)
Professionals	4 (0.6)
**Age, years, **Median (range)	69 (50–94)
**Education, years, **Median (range)	6 (0–22)
**Medical History**	
**Cardiovascular disease**	446 (71.5)
Myocardial	123 (19.7)
Hypertension	420 (67.3)
Peripheral vascular	64 (10.3)
**Pulmonary disease**	39 (6.3)
**Gastrointestinal disease**	253 (40.5)
Chronic Gastro-duodenitis/ulcer disease	225 (36.1)
Others: inflammatory bowel, liver disease etc.	49 (7.9)
**Genito-urinary disease**	47 (7.5)
Renal disease with renal insufficiency	40 (6.4)
Prostate disease	7 (1.1)
**Musculoskeletal and joint disorders**	597 (95.7)
Osteoarthritis	565 (90.5)
Rheumatoid arthritis	16 (2.6)
Gout	11 (1.8)
**Endocrine, alimentary and metabolic disease**	313 (50.2)
Diabetes mellitus	184 (29.5)
Thyroid gland disease	22 (3.5)
Hyperlipidemia	155 (24.8)
Obesity	39 (6.3)
**Neurological disease**	51 (8.2)
**Mental disease**	33 (5.2)
**Hematological disease**	17 (2.7)
**Ophthalmological disease**	26 (4.2)
**Dermatological disease**	14 (2.3)
**Infections and parasitic disease**	118 (18.9)
Respiratory infections	47 (7.5)
Gastrointestinal infections	6 (1.0)
Genito-urinary infections	43 (6.8)
Mycosis	28 (4.5)
**Trauma**	10 (1.6)
	**Mean **± SD*
**Disease number**	3.6 ± 1.5
Chronic disease	3.4 ± 1.5

Acute disease and trauma	0.2 ± 0.4

*SD: Standard deviation

**Table 2:** Prescribed drugs

Anatomical Therapeutic Chemical groups*	**Medications** n = 3739 **n (%)**	**Patients** n = 624 **n (%)**
**Alimentary tract and metabolism**	990 (26.5)	495 (79.3)
Drugs used in diabetes	250 (6.7)	179 (28.7)
Drugs for gastrointestinal disease**	437 (11.7)	332 (53.2)
Vitamins	236 (6.3)	209 (33.5)
Mineral supplements	88 (2.4)	87 (13.9)
**Blood and blood forming organs**	253 (6.8)	247 (39.6)
**Cardiovascular system**	929 (24.8)	470 (75.3)
**Dermatologicals**	81 (2.1)	66 (10.6)
**Genitourinary system and sex hormones**	20 (0.5)	20 (3.2)
**Systemic hormonal preparations**	29 (0.8)	27 (4.3)
**Antiinfectives for systemic use**	158 (4.2)	149 (23.9)
**Musculoskeletal system**	680 (18.2)	569 (91.2)
Non-steroidal anti-inflammatory drugs	611 (16.3)	560 (89.8)
Others drugs for the musculoskeletal system	69 (1.8)	61 (9.8)
**Nervous system**	380 (10.2)	298 (47.8)
Analgesics and antipyretics (Paracetamol)	229 (36.7)	229 (36.7)
Other nervous system drugs***	152 (4.1)	119 (19.1)
**Respiratory system**	117 (3.1)	81 (13.0)
**Sensory organs**	102 (2.7)	56 (9.0)
		
**Mean number of prescribed drugs**, Mean ± SD****		5.9 ± 2.5
**Number of prescribed drugs**		**n (%)**
2		22 (3.5)
3–4		168 (26.9)
5–6		210 (33.7)
≥ 7		224 (35.9)

*Anatomical Therapeutic Chemical classification system (ATC)

**A02–A09 in the ATC

***N01; N02A and C; N03–N07 in the ATC

****SD: Standard deviation

About 80% of patients had prescriptions for one or more combinations in the potential drug-drug interaction class. The most frequent drug interactions were type C, such as combinations of non-steroidal anti-inflammatory drugs (NSAIDs) with antihypertensive drugs (40.4%), and with low doses of acetylsalicylic acid (ASA) (34.0%). 3.8% of patients were prescribed drug combinations with interactions that should be avoided, and two patients were prescribed drugs with two potential type D interactions. Also, 400 patients (64.0%) had one or more potential drug-disease interactions of moderate clinical significance, given that the medicines prescribed could cause either light or moderate adverse effects. The most frequent were NSAIDs in patients with hypertension and/or chronic heart failure; β-blocking agents in patients with diabetes mellitus, and NSAIDs in patients with chronic renal failure (Table 3) When analyzing both classes of potential interactions we did not find statistically significant differences in patient gender, marital status or education.

**Table 3:** Potential drug-drug and drug-disease interactions

**Interactions**	**Patients** n = 624 **n (%)**
**Potential drug-drug interactions**	**492 (78.8)**
Number of potential drug-drug interactions*	
1	154 (24.7)
2	92 (14.7)
3–4	154 (24.7)
≥ 5	92 (14.7)
**Type D**	**24 (3.8)**
ACE* inhibitors + potassium-sparing diuretics (or potassium supplementation)	11 (1.8)
NSAID*+ Methotrexate	5 (0.8)
NSAID* +anticoagulants (or glucocorticoids)	5 (0.8)
β blocking agents + Verapamil (or Fluoxetin)	3 (0.4)
**Type C**	**468 (75.0)**
ACE* inhibitors + NSAID*	252 (40.4)
NSAID*+ low dose ASA*	212 (34.0)
NSAID*+ sulfonylureas	128 (20.5)
ACE* inhibitors + low dose ASA*	130 (20.8)
β blocking agents + NSAID*	107 (17.1)
NSAID* + Diuretics	105 (16.8)
ACE* inhibitors + glybenclamide	68 (10.9)
NSAID*+ other NSAID*	51 (8.2)
ACE* inhibitors + thiazide diuretics	47 (7.6)
Pravastatin + bezafibrate	35 (5.6)
Pentoxiyilline + antihypertensives	37 (5.9)
Metformin+ ranitidine	40 (6.5)
Glibenclamide + antimycotics or cotrimoxazole	24 (3.9)
Furosemide + ACE* inhibitors	21 (3.4)
ACE* inhibitors + antiacids	19 (3.1)
Alendronate + NSAIDs*	18 (2.9)
Insulin + antihypertensive	18 (2.9)
Pentoxifylline + hypoglycemic agents	14 (2.2)
Acetaminophen + carbamazepin	14 (2.2)
Ranitidine + azole type of antimycotics	11 (1.8)
Others	61 (9.4)
**Potential drug-disease interactions**	400 (64.1)
**Number of potential drug-disease interactions**	
1	346 (55.4)
≥ 2	54 (8.7)
NSAIDs* in patients with hypertension and/or chronic heart failure	384 (61.5)
β blocking agents in patients with diabetes mellitus	31 (5.0)
NSAIDs* in patients with chronic renal insufficiency	18 (2.9)
NSAIDs* in patients with previous peptic ulcer	8 (1.3)
Thiazides diuretics in patients with gout	7 (1.1)
Metformin in patients with chronic renal insufficiency or congestive heart failure	6 (1.0)
β blocking agents in patients with peripheral vascular disease	4 (0.6)
β blocking agents in patients with asthma/COPD*	3 (0.5)
Others	5 (1.0)

*ACE: angiotensin-converting enzyme; ASA: acetylsalicylic acid; COPD: chronic obstructive pulmonary disease; NSAID: Non-steroidal anti-inflammatory drugs.

Table 4 shows bivariate analysis of the relationship between the characteristics of patient and prescription, and potential drug-drug interactions. The variables found significant (p < 0.05) were, patient older than sixty years, 3 or more diseases, cardiovascular disease, endocrine, alimentary and metabolic disease and receiving five or more medicines.

**Table 4:** Relationship between patient's and prescription's characteristics and potential drug-drug interactions

**Variables**	**Patients without potential drug-drug interactions in their prescription** **n = 132**	**Patients with at least one potential drug-drug interactions in their prescription** **n = 492**
Gender (female)	105 (79.5)	386 (78.5)
Civil status (single)	61 (46.2)	259 (52.6)
Did not work	102 (77.3)	416 (84.6)
Literacy (only elementary school o less)	67 (54.9)	266 (62.9)
Patient age ≥ 60*	87 (65.9)	406 (82.5)
Number of disease ≥ 3*	73 (55.3)	396 (80.5)
Cardiovascular disease*	43 (32.6)	403 (81.9)
Musculoskeletal and joint disorders	126 (95.5)	471(95.7)
Gastrointestinal disease	48 (36.4)	205 (41.7)
Endocrine, alimentary and metabolic disease*	38 (28.8)	275 (55.9)
Pulmonary disease	8 (6.0)	31 (6.3)
Mental disease	12 (9.1)	21 (4.3)
Neurological disease	11 (8.3)	40 (8.1)
Infections and parasitic disease	30 (22.7)	88 (17.9)
Number of medicines ≥ 5*	50 (37.9)	383 (77.8)

*p < 0.05

The factors significantly associated with having one or more potential drug-drug interactions in the logistic regression model included: taking five or more medicines (adjusted odds ratio (aOR): 4.34, 95%CI: 2.76–6.83), patient age of 60 years or older (aOR: 1.66, 95% CI: 1.01–2.74) and suffering from cardiovascular diseases (aOR: 7.26, 95% CI: 4.61–11.44) (Table 5); the adjustment variables were: number of disease ≥ 3 and endocrine, alimentary and metabolic disease.

**Table 5:** Factors related to the potential drug-drug interactions

**Variables**	**Odds ratio**	**95% Confidence intervals**	**Adjusted Odds Ratio**	**95% Confidence intervals**
Patient age ≥ 60	2.44*	1.59 – 3.75	1.66*	1.01 – 2.74
Cardiovascular disease	9.37*	6.09 – 14.41	7.26*	4.61 – 11.44
Number of medicines ≥ 5	5.76*	3.82 – 8.69	4.34*	2.76 – 6.83

Adjustment variables: number of disease ≥ 3 and endocrine, alimentary and metabolic disease.

Goodness of fit > 0.05

*P < 0.05

The bivariate analysis of the factors associated with drug-disease interaction did not show any statistically significant association; therefore, a logistic regression analysis was not performed for these interactions.

## Discussion

Various studies have shown that potential drug-drug and drug disease interactions are frequent when patients receive multiple prescriptions. This is true for both ambulatory and hospitalized patients, and, in many cases, causes adverse effects and changes in therapeutic efficacies of the combined medicines, with consequent poor control of the diseases under treatment [1-4,6-12].

In the present study, we found that the frequency of potential drug-drug interactions in prescriptions of family doctors working in primary care clinics in Mexico City was almost 80.0%; this is higher than the frequency in Europe [4] (46.0%) and in the United States [1] (25.0%) in ambulatory patients over 59 years of age. The rates we found may be unique for the sample and may not be fully representative of Mexican population situation. The higher prevalence of potential drug-drug and drug-disease interactions in this study compared to others studies is likely attributable to the characteristics of the study sample (older adults with very high prevalence – nearly 90.0% – of NSAID utilization) among other reasons.

The frequency of type D interactions (which should be avoided) was smaller (3.8%) in our work, when compared with other studies (the type D frequency was 10.0% in the European study) [4]. The three combinations with drug-drug potential interactions that were found most frequently in our work are among those reported by Bjorkman [4]. These drug interactions are of type C and, without dose adjustment and patient monitoring, such interactions may antagonize drug effects on vascular tone and may result in increases in blood pressure (angiotensin-converting enzyme (ACE) inhibitors + NSAIDs, ACE inhibitors + low doses of ASA) [20] or may increase gastrointestinal adverse effects (NSAIDs + low doses of ASA) [21]. These finding coincide with the literature review performed by Becker et al., in which it was reported that drugs most often responsible for hospital admissions were NSAIDs and cardiovascular drugs, and the most common causes for such admissions were gastrointestinal tract bleeding and hyper- or hypotension [11].

Other authors have reported that both types C and D show similar hospitalization frequencies [10]. Therefore, medical doctors must be familiar with both interaction types.

We found that not only potential drug-drug, but also drug-disease interactions, were frequent in prescriptions. The frequency of the latter (64.0%) found in the present work is greater than reported in either hospitalized patients [2] or ambulatory patients [3].

The most frequent were interactions involving NSAIDs that were prescribed to patients with hypertension and/or chronic heart failure, and prescriptions of NSAIDs and ACE inhibitors. Although this finding was influenced by patient inclusion criteria, it shows that the flaws in the knowledge of prescribers regarding interactions of this group of drugs. In such cases, other therapeutic options should be considered to avoid potential drug interactions, like using paracetamol to manage osteoarthritis in patients with hypertension being treated with ACE inhibitors.

In general, our findings agree with other studies reporting that the risk of potential drug-drug interactions increases with each new prescription issued and with the aging of the patient [2,3,6-8]. In line with this, we found that patient age of 60 years or older and taking 5 or more medicines increases the risk of such potential interactions. Polypharmacy is an important problem in older people that has been reported as a frequent event all over the world [3,4,22,23]. In our sample, the patients took an average of 5.9 drugs, and those 60 years or older took an average of 6.1 drugs (37.3% of them took 7 or more medicines).

Within the context of this study, there exists limited published local information regarding the average number of drugs that a patient older than fifty years gets prescribed. Previous studies have been carried out in specific groups of older patients, such as community-dwelling elderly hospitalized due to inappropriate drug prescriptions [24], in nursing home residents [25] and in patients with hypertension [26]. For example, in hospitalized elderly patients, the average number of drugs consumed was 6.0 [24], and in nursing home residents was 2.8 [25], and in the study addressing patients with hypertension [26] aged 60 years and older, only 1.9% were taking three or more hypertensive drugs; yet, in this study the consumption of other drugs was not analyzed. Further studies should be advisable to gain in depth knowledge about the average number of drugs that family doctors prescribe to the elderly in Mexico.

Furthermore, we found that having a cardiovascular disease increases seven-fold the risk of potential drug-disease interactions. This means, as has been recommended in prior studies from other countries, that doctors need to pay more attention to drug prescription and patient monitoring when treating older individuals [2,3,6,7] also, the patients with cardiovascular diseases deserve more attention. It is necessary to consider interventions to reduce the drug interaction problem. Computer-based access to information on all prescriptions dispensed, and automated doctor alerts on the most frequent potential drug interactions encountered, would be most helpful. These tools are effective in reducing inappropriate prescriptions, and doctor acceptance in other populations has been reported [27,28]. Alternatives such as programs of continuous medical education, or pharmaceutical support, may also be considered. It has been found, however, that even pharmacists cannot detect all potential drug interactions because their number rises dramatically as the number of medicines prescribed increases [29].

Among the limitations of this study is that it only permits an approximation to problem of drug interactions in family medicine practice. The patients' group studied was very limited (all patients had non-malignant pain syndrome). We believe that some drug interactions may be more frequent in such patients. For example, we found that the most frequent potential drug-disease interaction involved the use of NSAIDs in patients with hypertension. In patients with acute or infectious disease is possible that other interactions would be found more frequently. Also, in this cross-sectional study we did not determine any possible relationship between drug-drug nor drug-disease interactions and the health status of the population studied.

It is possible to conclude that the high frequency of prescription of drugs with potential drug interactions is common in primary care level; the easiest way to reduce the frequency of them is to decrease the number of medicines prescribed. Nevertheless, sometimes it is difficult to reduce the number of drugs prescribed for patients with multiple chronic conditions; therefore, to lower the frequency of potential interactions it could be necessary to make a careful selection of therapeutic alternatives, and in cases without other options, patients should be continuously monitored to identify adverse events.

## Competing interests

The author(s) declare that they have no competing interests.

## Authors' contributions

SVD, HRM contributed in the conception and design of the study, literature review and statistical analysis. LPTA reviewed for important intellectual content. MSO contributed in data management. All authors participated in the interpretation of data and read and approved the final version to be published.

## Pre-publication history

The pre-publication history for this paper can be accessed here:



## References

[B1] Costa AJ (1991). Potential drug interactions in an ambulatory geriatric population. Fam Pract.

[B2] Lindblad CI, Artz MB, Pieper CF, Sloane RJ, Hajjar ER, Ruby CM, Schmader KE, Nalón JT (2005). Potential drug-disease interactions in frail, hospitalized elderly veterans. Ann Pharmacother.

[B3] Zhan C, Correa-de-Araujo R, Bierman AS, Sangl J, Miller MR, Wickizer SW, Stryer D (2005). Suboptimal prescribing in elderly outpatients: potentially harmful drug-drug and drug-disease combinations. J Am Geriatr Soc.

[B4] Bjorkman IK, Fastbom J, Schmidt IK, Bernsten CB (2002). Pharmaceutical Care of the Elderly in Europe Research (PEER) Group. Drug-drug interactions in the elderly. Ann Pharmacother.

[B5] Charlson ME, Pompei P, Ales KL, McKenzie CB (1987). A new method for classifying prognosis in longitudinal studies: development and validation. J Chron Dis.

[B6] Janchawee B, Wongpoowarak W, Owatranporn T, Chongsuvivatwong V (2005). Pharmacoepidemiologic study of potential drug interactions in outpatients of a university hospital in Thailand. J Clin Pharm Ther.

[B7] Cruciol-Souza JM, Thomson JC (2006). Prevalence of potential drug-drug interactions and its associated factors in a Brazilian teaching hospital. Pharm Pharm Sci.

[B8] Merlo J, Liedholm H, Lindblad U, Bjorck-Linne A, Falt J, Lindberg G, Melander A (2001). Prescriptions with potential drug interactions dispensed at Swedish pharmacies in January 1999: cross sectional study. BMJ.

[B9] Stanton LA, Peterson GM, Rumble RH, Cooper GM, Polack AE (1994). Drug related admissions to an Australian hospital. J Clin Pharm Ther.

[B10] Doucet J, Chassagne P, Trivalle C, Landrin I, Pauty MD, Kadri N, Menard JF, Bercoff E (1996). Drug-drug interactions related to hospital admissions in older adults: a prospective study of 1000 patients. J Am Geriatr Soc.

[B11] Becker ML, Kallewaard M, Caspers PW, Visser LE, Leufkens HG, Stricker BH (2007). Hospitalizations and emergency department visits due to drug-drug interactions: a literature review. Pharmacoepidemiol Drug Saf.

[B12] Seymour RM, Routledge PA (1998). Important drug-drug interactions in the elderly. Drugs Aging.

[B13] Viramontes Madrid JL, Jerjes Sánchez C, Pelaez Ballestas I, Hernández Garduño AG, Aguilar Chiu A (2002). Risk of drug interactions. Drug combinations that are associated to arrhythmia ventricular. Rev Invest Clín.

[B14] Campos-Garza JF, Aquino-Arteaga A, Uc-Morales DN, Herrera-Huerta EV, Velásquez-Hernández F, Hernández Cruz R (2006). Detection of drug-drug interactions in the Internal Medicine Service of the regional general hospital of Orizaba Veracruz. Journal of Health Publishes and Nutrition.

[B15] Zenón TD, López Guzmán JA, Roldán de la O I, Alvarado JA, Villalobos AS, d'Hyver de las Deses C (2005). Inappropriate prescriptions in the elderly: classification proposal. Med Int Mex.

[B16] World Health Organization (1989). International Statistical Classification of Diseases and Related Health Problems : 10th revision (ICD-10). Geneva.

[B17] ATC Index with DDDs (2002). January 2002: WHO Collaborating Centre for Drug statistics Methodology. Oslo.

[B18] Thomson MICROMEDEX (2006). MICROMEDEX(R) Healthcare Series Drug Reference Guides. Drug information for the health care professional. Vol 124 expires 6.

[B19] FASS (Pharmaceutical Specialities in Sweden) (1997). Stockholm: INFO Lakemedelsinformation AB (Drug information). http://www.fass.se.

[B20] Yost JH, Morgan CJ (1994). Cardiovascular effects of NSAISDs. J Musculoskel Med.

[B21] Sorensen HT, Mellemkjaer L, Blot WJ, Nielsen GL, Steffensen FH, McLaughlin JK, Olsen JH (2000). Risk of upper gastrointestinal bleeding associated with use of low-dose aspirin. Am J Gastroenterol.

[B22] Kaufman DW, Kelly JP, Rosenberg L, Anderson TE, Mitchell AA (2002). Recent patterns of medication use in the ambulatory adult population of the United States: the Slone survey. JAMA.

[B23] Oscanoa T, Lira G (2005). Medicines prescription quality for geriatric care. An Fac Med Latina.

[B24] Zenón TG, López Guzmán JA, Roldan de la O I, Alvarado JA, Villalobos JA, d'Hyver de las Deses C (2005). Inappropriate drugs in elderly: classification proposal. Med Intern Mex.

[B25] Pérez-Guillé G, Camacho-Vieyra A, Toledo-López A, Guillé-Pérez A, Flores-Pérez J, Rodríguez-Pérez R, Juárez-Olguín H, Lares-Asseff I (2000). Patterns of drug consumption in relation with the pathologies of elderly Mexican subjects resident in nursing homes. J Pharm Pharm Sci.

[B26] Garcia-Pena C, Thorogood M, Reyes S, Salmeron-Castro J, Duran C (2001). The prevalence and treatment of hypertension in the elderly population of the Mexican Institute of Social Security. Salud Publica Mex.

[B27] Tamblyn R, Huang A, Perreault R, Jacques A, Roy D, Hanley J, McLeod P, Laprise R (2003). The medical office of the 21st century (MOXXI): effectiveness of computerized decision-making support in reducing inappropriate prescribing in primary care. CMAJ.

[B28] Shah NR, Seger AC, Seger DL, Fiskio JM, Kuperman GJ, Blumenfeld B, Recklet EG, Bates DW, Gandhi TK (2006). Improving acceptance of computerized prescribing alerts in ambulatory care. J Am Med Inform Assoc.

[B29] Weideman RA, Bernstein IH, McKinney WP (1999). Pharmacist recognition of potential drug interactions. Am J Health-Syst Pharm.

